# Recent Caffeine Drinking Associates with Cognitive Function in the UK Biobank

**DOI:** 10.3390/nu12071969

**Published:** 2020-07-02

**Authors:** Marilyn C. Cornelis, Sandra Weintraub, Martha Clare Morris

**Affiliations:** 1 Department of Preventive Medicine, Feinberg School of Medicine, Northwestern University, Chicago, IL 60611, USA; 2 Mesulam Center for Cognitive Neurology and Alzheimer’s disease, Feinberg School of Medicine, Northwestern University, Chicago, IL 60611, USA; sweintraub@northwestern.edu; 3 Department of Psychiatry and Behavioral Sciences, Feinberg School of Medicine, Northwestern University, Chicago, IL 60611, USA; 4 Rush Institute for Healthy Aging, Rush University, Chicago, IL 60611, USA; Martha_Morris@rush.edu

**Keywords:** caffeine, acute, genotype, reaction time, cognition, memory, cohort

## Abstract

Clinical evidence points to the premise that caffeine may benefit cognition, but whether these findings extend to real life settings and amidst factors that impact caffeine metabolism is unclear. The aim of this study was to investigate the impact of recent caffeine drinking on cognitive ability while additionally accounting for lifestyle and genetic factors that impact caffeine metabolism. We included up to 434,900 UK Biobank participants aged 37–73 years, recruited in 2006–2010, who provided biological samples and completed touchscreen questionnaires regarding sociodemographic factors, medical history, lifestyle, and diet. Recent caffeine drinking (yes/no in the last hour) was recorded during a physical assessment. Participants completed at least one of four self-administered cognitive function tests using the touchscreen system: prospective memory (PM), pairs matching (Pairs), fluid intelligence (FI), and reaction time (RT). Multivariable regressions were used to examine the association between recent caffeine drinking and cognition test scores. We also tested interactions between recent caffeine drinking and a genetic caffeine-metabolism score (CMS) on cognitive function. Among white participants, recent caffeine drinking was associated with higher performance on RT but lower performance on FI, Pairs, and PM (*p* ≤ 0.004). Similar directions of associations for FI (*p* = 0.09), Pairs (*p* = 0.03), and PM (*p* = 0.34) were observed among non-white participants. No significant and consistent effect modification by age, sex, smoking, test time, habitual caffeine intake, or CMS was observed. Caffeine consumed shortly before tasks requiring shorter reaction times may improve task performance. Potential impairments in memory and reasoning tasks with recent caffeine drinking warrant further study.

## 1. Introduction

Readily available from coffee, tea, and other food stuffs, caffeine is the most widely consumed psychostimulant in the world [1]. Much evidence supports a role of caffeine in enhancing cognitive function. Tasks involving cognitive functions such as attention, vigilance, and reaction time are particularly responsive to caffeine [2,3,4,5,6]. Some argue that most of these effects reflect restoration of cognitive function impaired by caffeine withdrawal [7,8]. Caffeine withdrawal is any withdrawal syndrome that occurs after abrupt cessation or reduction of caffeine intake and is characterized most often by headache, but also drowsiness, depression, low energy, irritability, difficulty concentrating, and flu-like symptoms [9,10]. Indeed, in acute clinical studies of caffeine and cognition, habitual caffeine consumers are often asked to abstain from caffeine overnight or longer, which may induce withdrawal symptoms [11,12,13]. Others, however, reject this hypothesis since some studies report similar performance improvements for habitual consumers and non-consumers of caffeine or improved performance in non-withdrawn consumers [5,14].

The typical clinical caffeine study design differs from the real-world, where individuals may drink caffeine regularly throughout the day, maintaining consistent plasma caffeine levels and rarely experiencing overt withdrawal symptoms. Indeed, evidence suggests that individuals are capable of self-regulating caffeine intake to achieve their optimal level of arousal [15,16]. Furthermore, genetic and lifestyle factors contributing to known between-person variation in caffeine metabolism or sensitivity are rarely considered in acute clinical studies [17,18,19,20,21].

The UK Biobank is a large population cohort of adults who underwent medical, sociodemographic, lifestyle, and cognitive assessment. The current study leverages this valuable resource to investigate the impact of recent caffeine intake on cognitive ability while additionally accounting for lifestyle and genetic factors that impact caffeine metabolism. We hypothesized that recent caffeine intake would improve cognitive performance overall, but particularly among those with a genetic predisposition for slower caffeine metabolism.

## 2. Subjects and Methods

### 2.1. Participants and Protocol Overview

In 2006–2010, the UK Biobank recruited over 502,633 participants aged 37–73 years at 22 centers across England, Wales, and Scotland [22]. At each center, participants completed touchscreen questionnaires on sociodemographic factors, lifestyle, and medical history while seated at a desktop computer workstation. A series of cognitive function tests were also administered via touchscreen and required about 15 min to complete [23]. The room was large enough to accommodate 10–12 workstations and to provide each participant with privacy by spacing and partitions. This questionnaire period was followed by an in-person questionnaire, physical assessment, and biospecimen collection period. Detailed study methods are provided in the Supplementary Materials and the order of operations are presented in Supplementary Table S1. This study was covered by the generic ethical approval for UK Biobank studies from the National Research Ethics Service Committee, North West-Haydock (approval letter dated 17 June 2011, Ref 11/NW/0382), and all study procedures were performed in accordance with the World Medical Association Declaration of Helsinki ethical principles for medical research.

### 2.2. Cognitive Function Testing

Prospective Memory (PM) Test: This test was added part-way through the baseline assessment period. Participants were given the following instructions: “At the end of the games (other cognitive function tests), we will show you four colored symbols and ask you to touch the blue square. However, to test your memory, we want you to actually touch the orange circle instead.” Participants were scored as zero or one, depending on whether they completed the task on the first attempt or not. Cohen’s kappa = 0.36 for PM has been reported elsewhere [24].

Pairs Matching (Pairs) Test: For this episodic visual memory test, participants were shown 6 pairs of cards for 3 s, which were then turned over. Participants were asked to identify the matching pairs and the total number of errors made during this task was recorded. We restricted our analyses to individuals who finished the test and log (+1) transformed the number of errors for the analysis.

Fluid Intelligence (FI) Test: This test for verbal-numerical reasoning was added part-way through the baseline assessment period and completed at the assessment centers. Participants were presented with 13 verbal logic/reasoning-type questions and had to answer as many as they could within 2 min. One question, for example, was “Age is to years as height is to?” with the following options to choose from: long, deep, top, meters, tall, do not know, prefer not to answer. Incorrect or unattempted questions were scored as zero. The total number of correct answers (max 13) was used for our analysis. The Cronbach alpha coefficient for these items has been reported elsewhere as 0.62 [25].

Reaction Time (RT) Test: For this measure of simple processing speed, participants completed a timed test of symbol matching at the assessment centers. The score on this task was the mean response time in milliseconds (ms) across 4 trials which contained matching pairs. Potential outliers were truncated to 100 (min) or 1000 (max) ms. Cronbach’s alpha for this task has previously been reported as 0.85 [25].

### 2.3. Recent Caffeine Drinking

Participants were not asked to fast prior to the assessment visit. At the reception station, participants were advised that water would be available at all times during the visit and that a urine sample will be sought at the end of the visit. About 20 min after cognitive function testing, participants completed a spirometry test. The screening questionnaire for spirometry testing asked whether they had drank caffeine within the last hour. Participants replied Yes or No. During blood sample collection, after spirometry testing, participants were asked to provide “Time since last meal or drink (except plain water)”. Tea and coffee were available only at the end of the visit.

### 2.4. Habitual Caffeine Consumption and other Covariates

Information on habitual diet and several covariates functioning as potential confounders in observation analysis of caffeine and cognitive function were also collected during the UK Biobank assessment, as described in detail previously [22,26]. The touchscreen questionnaire included a dietary assessment of a range of common food and drink items. Information on caffeine containing items was limited to that of coffee and tea. For coffee intake, participants were asked “How many cups of coffee do you drink each day” (include decaffeinated coffee)”. Participants either selected the number of cups, “less than one”, “do not know”, or “prefer not to answer”. Participants who reported drinking coffee were then asked, “What type of coffee do you usually drink?” and were able to select one of three responses: “Decaffeinated coffee (any type)”, “Instant coffee”, or “Ground coffee (include espresso, filter, etc.)”. For participants reporting more than 15 cups/day, we re-assigned their intake to 15 cups/day. A similar question was asked about tea (include black and green tea), but no additional details on type of tea was collected. We estimated total caffeine (mg/day) from (regular) coffee and tea by assigning each cup 75 mg and 40 mg of caffeine, respectively [27].

For the current analysis, we also considered baseline smoking status, Townsend deprivation index (higher scores higher deprivation), education, income, home-ownership, physical activity, ethnicity, employment status, self-rated health, water intake, alcohol intake, fish intake, red meat intake, fruit intake, and vegetable intake. The time when cognitive function testing took place at the center was recorded for only the PM test and was therefore also used as a proxy time for FI, Pairs, and RT.

### 2.5. Follow-Up Assessments

Subsets of participants completing the baseline visit returned to the assessment centers for up to two follow-up assessments. The first follow-up took place from 2012–2013 and included 20,346 participants, all living within 35 km of the Stockport Biobank coordinating center, but only 84 reported recent caffeine intake. The same region was targeted for a second follow-up in 2014, and at the time of the current study, sufficient data from 17,780 white and 578 non-white participants was available, and 3603 and 90 reported recent caffeine intake, respectively.

### 2.6. Genetic Data

All UK Biobank participants were genotyped using genome-wide arrays. The initial 50,000 participants were genotyped using the Affymetrix UK BiLEVE Axiom array, while the remaining were genotyped using the Affymetrix UK Biobank Axiom array. The two arrays were extremely similar and can be imputed together. Quality control (QC) and imputation to the Haplotype Reference Consortiumv1.1 and UK10K reference panels were performed centrally by the Wellcome Trust Centre for Human Genetics, as described elsewhere [28]. We excluded sample outliers based on heterozygosity and missingness, participants with sex discrepancies between the self-reported and X-chromosome heterozygosity, and those potentially related to other participants, based on kinship coefficients estimated from genetic data using the software *KING* [28,29]. For the latter, one participant of each parent-child, sibling, and first-cousin pair was excluded. A genetic ‘caffeine metabolism” score (CMS_G_) was derived using two single-nucleotide polymorphisms (SNPs), presenting with the largest effect sizes in a genome-wide association study (GWAS) of caffeine metabolites: rs2472297 (near *CYP1A2*) and rs6968554 (near *AHR*) [21], by summing the number of alleles multiplied by their beta coefficients. The latter were estimated by *z*/ (sqrt (*p* (1-*p*))), where *z* is the SNP *z*-score for the paraxanthine to caffeine ratio and *p* is the SNP minor allele frequency. Estimated beta coefficients for rs6968554 and rs2472297 were 17.58 and 21.58, respectively. CMS_G_ was calibrated such that it ranged from 0 to 4, with higher scores predicting faster caffeine metabolism. We conducted a separate analysis of rs762551 (CYP1A2*1F) since this SNP has been examined previously for interactions with coffee and disease outcomes [30]. *APOE* carriers (ε4+) and non-carriers (ε4−) were defined using genotyped or imputed genotypes for SNPs rs429358 and rs7412. We limited the genetic analysis to unrelated individuals who self-report as “British” and who have very similar ancestral backgrounds based on results of principal component analysis.

### 2.7. Statistical Analysis

All statistical analyses were performed using SAS software, release 9.4 (SAS Institute Inc, Cary, NC, USA). A total of 454,020 participants had information on recent caffeine drinking and completed at least one of the cognitive function tests at baseline. Because some cognitive tasks were added at different stages of baseline assessment, the number of participants varies across tests (see Supplementary Table S1 for detailed sample sizes). We excluded 19,120 who self-reported neurological disease at baseline follow-up that could directly affect cognitive function, leaving up to 434,900 participants for analysis [24]. Main analysis proceeded separately for self-described white (*N* = 410,183) and non-white participants (*N* = 24,717) to facilitate comparison to the results of genetic analysis, which were limited to white British ancestry.

We examined the association between recent caffeine drinking (yes versus no) and cognitive test scores using linear (FI, RT, Pairs tests) or logistic (PM test) regression, adjusting for age, sex, and ethnicity (for non-white analysis only) (model 1). In multivariable regressions, we further adjusted for smoking (never, past, current: <10, 10 to 19, 20+ cigs/day), fasting status (0–1, 3 to 4, 5+ h), Townsend deprivation index (quartiles), education (7 categories), income (4 levels), home-ownership (yes, no), physical activity (quartiles of moderate/vigorous activity minutes/week), employment status (employed, retired, other), waist-to-hip ratio, self-reported health (excellent, good, fair, poor), and intakes of water, alcohol, fish, red meat, fruits, and vegetables (quartiles of servings/week) (model 2). Missing indicator variables were constructed to maximize sample size but were required for <1% of the sample (see Table S1). A third multivariable regression model was further adjusted for habitual coffee (7 categories: none, <1, 1, 2–3, 4–5, 6–7, and ≥8 cups/day) and tea intake (7 categories) (model 3). Associations with *p*-values < 0.05 were considered statistically significant. In sensitivity analysis, we further adjusted models separately for exam time, diabetes, and hypertension status.

We tested interactions with age (<55 or ≥55 years of age), sex, habitual caffeine consumption (<100 or 100+ mg/day), education (university/college degree or less), smoking (never, past, or current), fasting status (three time intervals), exam time (before 12 pm or after 12 pm), and *APOE* ε4 carrier status by including in multivariate regression models the cross-product term of recent caffeine drinking with the interacting variable. The time when cognitive function testing took place at the center was recorded for only the PM test (introduced part-way through recruitment and was therefore also used as a proxy time for FI, Pairs, and RT). Given that the CMS_G_ SNPs are related to both caffeine drinking behavior and circulating levels of caffeine (i.e., metabolism), we performed analysis stratified by CMS_G_ (0–1, 1–2, or 3–4 allelic metabolism score, defined using 2 SNPs, *AHR*, and *CYP1A2*), in addition to formal gene–diet interaction testing. Significant interactions were defined as *p* < 0.006, after applying a correction for 9 potential modifiers.

In exploratory analysis, we considered stratified combinations of age, exam time, habitual caffeine intake, and CMS_G_. For the white subset of participants with follow-up assessment in 2014, we also performed a synthetic cross-over trial analysis with the 1070 (FI) to 3307 (RT) participants reporting recent caffeine intake (“treatment”) in the first assessment, but no recent caffeine intake (“no treatment”) in the second assessment (period 1) and the [31] (FI) to 190 (RT) participants receiving the “treatment” and “no treatment” in the reverse order (period 2).

## 3. Results

### 3.1. Participant Characteristics

Descriptive characteristics of participants who reported recent caffeine drinking and those who did not are presented in Table 1. Among both white and non-white participants, recent caffeine drinkers were younger and more likely to be male, current smokers, have a higher Townsend deprivation score (more deprivation), a higher income, a university or college degree, and to consume more tea and coffee (particularly of the regular ground type) than non-recent caffeine drinkers. They were also more likely to attend the assessment center earlier and to be fasting for a shorter duration. Characteristics of the unrelated (white) British Ancestry subgroup according CMS_G_ are presented in Supplementary Table S2. As anticipated, CMS_G_ was significantly associated with habitual coffee and tea consumption (*p* < 0.0001), with higher CMS_G_ associated with higher intakes. Each SNP contributing to CMS_G_ as well as rs762551 was also associated with habitual coffee (*p* < 0.0001) and tea (*p* < 0.0001) consumption. Those with higher CMS_G_ were also more likely to report recent caffeine drinking. Body mass index, waist-to-hip ratio, number of alcohol drinks, and servings of meat and fruit also significantly differed by CMS_G_, but absolute differences were very small.

### 3.2. Recent Caffeine Drinking and Cognitive Function

Among white participants, recent caffeine drinking was associated with significantly lower performance on FI, Pairs, and PM, but better performance on RT in either crude or fully adjusted models (*p* ≤ 0.004, Table 2). Similar results were observed when further adjusting for disease status or when adjusting for habitual regular coffee (instead of total coffee) intake. When adjusting for exam time (available for a subset of participants), the effect estimates for RT were noticeably weaker (β = −2.90, model 3) but nevertheless significant (*p* = 0.009). A similar pattern of results was observed among non-white participants, with the exception of RT (Table 3). RT increased with recent caffeine drinking in non-white participants but differences were not significant.

### 3.3. Interactions with Demographic and Lifestyle Factors, Fasting Time, Exam Time, and APOE

For white and non-white participants, the caffeine-cognition test associations reported above were similar across strata defined by age, sex, education, habitual caffeine intake, fasting status, exam time, and *APOE* genotype (Tables S3–S9, and data not shown, *p* ≥ 0.02 for interactions). A nominally significant smoking × recent caffeine interaction (*p* = 0.007) was observed in non-white participants: recent caffeine intake lengthened RT among past (*p* = 0.06) and current (*p* = 0.02) smokers but not among never smokers (*p* = 0.46) (Supplementary Table S10, *p* = 0.007 for interaction). This interaction was not supported by formal interaction testing *(p* > 0.10) or smoking stratified analysis among white participants. We additionally explored the association between recent caffeine drinking and cognitive function in subgroups of white participants defined by both habitual caffeine consumption and exam time (Table S11), and by both age and exam time (Table S12), but no evidence for effect modification was observed.

### 3.4. CMS_G_ Main Effects and Interactions with Recent Caffeine Drinking on Cognitive Function

CMS_G_ was associated with PM: higher CMS_G_ was associated with better PM performance but only when adjusting for coffee and tea intake (*p* = 0.03). *AHR* (rs6968554) genotype was significantly associated with RT with or without adjustment for habitual caffeine intake (all *p* < 0.007). Each rs6968554 allele previously linked to lower caffeine metabolite levels (i.e., faster caffeine metabolism) was associated with longer RT. SNPs near *CYP1A2* were not associated with cognitive test performance. Results were similar when adjusted for 20 PCs.

We observed no significant interactions between recent caffeine drinking and CMS_G_ (Table 4) or individual SNPs (Tables S13–S15) on cognitive function. Stratified analysis suggested that recent caffeine drinking was associated with increasing RT performance with increasing CMS_G_ score. The opposite pattern was observed for Pairs performance.

### 3.5. Synthetic Cross-Over Trial

We performed a synthetic cross-over analysis leveraging those participants returning to the assessment center in 2014 with discordant recent caffeine intake status from their first assessment. No significant period effects were observed for FI and Pairs tests (*p* > 0.34). Recent caffeine intake (treatment) improved FI (mean change (95% CI): 0.07 (−0.26, 0.40), *p* = 0.40) and Pairs (−0.02 (−0.07, 0.04, *p* = 0.34) performance but these changes (treatment effects) did not reach statistical significance. A significant period effect was observed for RT, whereby RT was shorter for period 1 than period 2 (mean change (95% CI): 48 (41–55), *p* < 0.0001). No significant treatment effect was observed for RT. For PM, significant period (*p* < 0.0001) and treatment (*p* < 0.0001) effects were observed. Recent caffeine intake improved PM performance but this was restricted to period 1, wherein overall PM performance was lower than that of period 2.

## 4. Discussion

Clinical studies generally report improved attention, vigilance, and reaction time with acute intakes of caffeine, while cognitive functions such as problem solving and memory are less responsive to caffeine [2,3,4,5,6,32,33]. How these clinical findings generalize to real-life settings where caffeine is widely available is unclear. The current study used a novel observational framework to investigate the relationship between recent (acute) caffeine drinking, defined as any within the hour, on cognitive function. Overall, recent caffeine intake improved reaction time among whites but decreased memory and reasoning performance in whites as well as non-whites. These findings were not significantly modified by environment factors, *APOE*, or genetic differences in caffeine metabolism.

Some argue that the acute effects of caffeine are largely due to reversal of withdrawal symptoms, which usually begin after 12 to 16 hours of acute caffeine abstinence and can persist for up to a week [7,9,34]. Efforts to address withdrawal reversal often involve comparing the effects of caffeine on cognition in habitual caffeine consumers and non-consumers or integrating a caffeine pretreatment stage [7]. However, individuals who do not habitually consume caffeine may be sensitive to the drug and, hence, avoid its use [35]. A caffeine procedure may also fail to fully reverse withdrawal for some caffeine consumers [7]. James and Rogers encourage studies in which performance and mood have been compared in the same participants who have experienced prolonged periods of caffeine and placebo, with the aim of effectively “washing out” the effects of caffeine tolerance and withdrawal, but these studies are demanding on participants and research resources [7] and are also subject to selection bias. An important strength of the current study is therefore the large natural experiment approach to a research question traditionally investigated in the highly controlled clinical setting with a few dozen participants. Moreover, evidence suggests that individuals are capable of self-regulating caffeine intake to achieve their optimal level of arousal while incurring minimal negative effects [15,16]. With this behavior in mind and given no particular guidelines concerning caffeine consumption or fasting, it is unlikely that participants visiting the assessment center were selected on caffeine consumption behavior, per se, or were experiencing any overt caffeine withdrawal symptoms.

To our knowledge, the only study comparable to ours is that by Lesk et al. [36], who asked 98 elderly participants of the Oxford Project to Investigate Memory and Aging (OPTIMA) about recent caffeine intake before cognitive testing. Information on all caffeine-containing foodstuffs consumed within 4 h of the cognitive testing was collected. Overall, recent caffeine intake (yes or no) was not associated with any measure of cognitive function. However, age × caffeine intake interactions were found for tests of episodic memory, semantic memory and processing speed, but not general cognition or working memory. Participants 80+ years of age performed significantly worse if they had consumed caffeine, and those 60–79 years of age performed significantly better if they had consumed caffeine [36]. The results of the current study were not modified by age. However, given the younger age of participants (37 to 73 years) and shorter caffeine exposure window (1 h) of the current study, we are unable to directly compare our findings to that of Lesk et al. [36].

After ingestion, caffeine is quickly absorbed from the gastrointestinal tract into the circulatory system [3,37]. The maximum plasma concentration of caffeine is reached after 30–60 min from consumption. Because evidence suggests that individuals are capable of self-regulating caffeine intake to achieve their optimal level of arousal [15,16], we hypothesized that if caffeine has any “net” effect on cognitive function, then ‘any’ amount of caffeine 1 hour prior to testing would improve testing performance. This hypothesis was supported by our findings for RT but not for other tests where we observed impaired performance with recent caffeine drinking. These findings suggest that caffeine does not influence general aspects of functioning but rather processes specific to each test, also observed in the clinical setting. Memory and reasoning tests have not generally been shown to benefit from acute caffeine intake but the impaired performance we observed on these tests was unexpected. Lesk et al. [36] reported lower performances on memory and other tests with recent caffeine intake but only among those 80+ years of age. They suggested the results may have been due to coffee intake increasing homocysteine levels, which negatively correlate with cognitive test performance in the elderly [38,39]. This explanation is unlikely to explain our results since Lesk et al. [36] reported improved performance on memory tests among those 60–79 years, an age interval that overlaps with the current study. It is possible that unmeasured factors correlating with recent caffeine drinking and that negatively impact processes specific to these tests confounded our analysis. Our synthetic cross-over study using participants with follow-up assessments did not confirm our overall findings. However, these efforts were challenged by period effects, likely attributable to age-related declines and learning effects that we previously reported for this population [40].

Caffeine’s half-life in humans ranges from 2 to a maximum of 12 h [41], mainly due to the interindividual variability in metabolism. Smoking, sex, heavy alcohol and caffeine intake, and genetic variation alter caffeine metabolism [17,18,19,20,21], and were factors we adjusted for or investigated for effect modification. Genetic factors were of particular interest given that they overcome some of the limitations of self-reported lifestyle factors [30]. Overall, we found no strong support for a modifying effect of CMS_G_ on associations between recent caffeine drinking and cognitive function that would be consistent with our hypothesis: recent caffeine drinking would improve cognition performance, particularly among individuals with CMS_G_, corresponding to slower caffeine metabolism. It is possible that the self-regulating behavior of caffeine drinking, a consequence of all factors contributing to individuals differences in metabolism, effectively washed-out any gene–caffeine interactions. The current study narrowed in on genetic factors related to caffeine metabolism which have previously been confirmed as being associated with caffeine intake behavior and circulating caffeine levels in GWAS. Physiologically relevant doses of caffeine block the A1 and A2a receptors and thus modulate the activity of the sympathetic nervous system by blocking the effects of adenosine [3]. Future studies of acute caffeine intake and cognitive function might additionally account for genetic variation in the adenosine receptor pathway.

A number of study limitations should be considered when interpreting the results. Recent caffeine drinking was defined simply as yes or no and could not be verified by a biomarker such as blood caffeine metabolite levels. No information on caffeine beverage source, amount, or preparation was recorded. Per study protocol, the average estimated time between reception and spirometry testing was 75 min. Individuals reporting drinking caffeine within an hour of spirometry testing might therefore have (i) taken less time to complete the full protocol than estimated and/or (ii) inaccurately estimated the time since caffeine was consumed—factors which would have contributed to study measurement error. Measures of habitual caffeine intake were derived from only coffee and tea. However, based on a subset of about 100,000 UK Biobank participants completing multiple on-line dietary records, the consumption of other caffeine sources such as soda and chocolate containing foods and beverages was low [42,43]. That being said, our findings might also be limited to coffee or tea sources of caffeine. Missing exam time for a large subset of participants limited our ability to further explore exam time as a confounder. The approach to data capture in the UK Biobank aimed to optimize the accuracy and completeness of the data collected with maximal efficiency. Thus, only a few and brief tests of cognition were selected. The UK Biobank is also not representative of the sampling population, with evidence of a ‘healthy volunteer’ selection bias [44], and thus, extrapolation of our findings to a more general population is limited. Finally, while the findings of the current study are statistically significant, the absolute differences in cognitive function scores are unlikely to be of broad practical significance. For example, we observed a 13 ms faster RT with recent caffeine drinking, and when adjusting this time for confounders, it was shortened to 5 ms. The importance of these differences might only be applicable to emergency situations.

Caffeine is the most widely consumed psychostimulant in the world [1]. Our findings suggest that caffeine consumed shortly before tasks requiring shorter reaction times may improve task performance. Potential impairments in memory and reasoning tasks with recent caffeine drinking warrant further study.

## Figures and Tables

**Table 1:** Baseline characteristics of UK Biobank participants according to recent caffeine drinking status ^1^.

Characteristic	Whites		Non-Whites	
	No	Yes	No	Yes
	*N* = 401,650	*N* = 8533	*N* = 24,152	*N* = 565
Mean (SD) age, years ^2^	56.6 (8.0)	54.6 (8.2)	52.6 (8.2)	51.0 (7.6)
Male	179,881 (44.8)	4110 (48.2)	11,126 (46.1)	272 (48.1)
Ethnicity ^2^				
Mixed, other, unknown			7775 (32.2)	233 (41.2)
Asian			8199 (34.0)	162 (28.7)
Black			6823 (28.3)	119 (21.1)
Chinese			1355 (5.6)	51 (9.0)
Smoking status ^2^				
Never	220,291 (54.9)	4132 (48.4)	16,496 (68.3)	315 (55.8)
Past	140,666 (35.0)	2921 (34.2)	4660 (19.3)	128 (22.7)
Current	40,693 (10.1)	1480 (17.3)	2999 (12.4)	122 (21.6)
Mean (SD) body mass index, kg/m^2^	27.3 (4.7)	27.4 (4.7)	27.8 (5.0)	27.4 (5.0)
Mean (SD) waist-to-hip ratio	0.87 (0.09)	0.87 (0.09)	0.88 (0.08)	0.87 (0.08)
Income				
<18,000	72,866 (18.1)	1536 (18.0)	5655 (23.4)	124 (22.0)
18,000–30,999	88,084 (21.9)	1771 (20.8)	4668 (19.3)	114 (19.7)
31,000–51,999	92,846 (23.1)	2057 (24.1)	4222 (17.5)	92 (16.3)
52,000–100,000	73,766 (18.4)	1636 (19.2)	2986 (12.4)	85 (15.0)
100,000+	19,650 (4.9)	540 (6.3)	858 (3.6)	20 (3.5)
Will not answer, missing	54,438 (13.6)	993 (11.6)	5763 (23.9)	133 (23.5)
Education				
None (listed), prefer not to answer	69,013 (17.2)	1415 (16.6)	4152 (17.2)	94 (16.5)
CSEs or equivalent	15,021 (3.7)	375 (4.4)	984 (4.1)	12 (2.1)
O levels/GCSEs or equivalent	53,379 (13.3)	1065 (12.5)	2474 (10.2)	54 (9.6)
A levels/AS levels or equivalent	22,409 (5.6)	447 (5.2)	1317 (5.5)	36 (6.4)
Other professional qualifications	48,708 (12.1)	922 (10.8)	2385 (9.9)	49 (8.7)
NVQ or HND or HNC or equivalent	62,073 (15.5)	1371 (16.1)	3222 (13.3)	69 (12.2)
College or University degree	131,047 (32.6)	2938 (34.4)	9618 (39.8)	252 (44.6)
Employment status ^2^				
currently employed	235,513 (58.6)	5662 (66.4)	15,505 (64.2)	368 (65.1)
retired	134,175 (33.4)	2118 (24.8)	3912 (16.2)	54 (9.6)
other/not reported	31,962 (8.0)	753 (8.8)	4735 (19.6)	143 (25.3)
Mean (SD) Townsend deprivation score	−1.52 (2.95)	−1.15 (3.17)	1.02 (3.57)	0.87 (3.56)
Home owner	362,769 (90.3)	7415 (86.9)	16,460 (68.2)	365 (66.4)
Mean (SD) moderate to vigorous physical activity, minutes/week	77 (96)	78 (105)	65 (94)	71 (113)
Hypertension ^2^	211,303 (52.6)	4193 (49.1)	12,169 (50.4)	249 (44.1)
Diabetes	17,938 (4.5)	377 (4.4)	2713 (11.2)	65 (11.5)
Self-reported overall health rating				
Excellent	71,289 (17.8)	1566 (18.5)	2692 (11.4)	81 (14.9)
Good	239,260 (59.8)	4973 (58.6)	12,707 (53.7)	280 (51.4)
Fair	76,656 (19.2)	1641 (19.3)	6735 (28.5)	145 (26.6)
Poor	13,115 (3.3)	309 (3.6)	1542 (6.5)	39 (7.2)
Mean (SD) alcohol drinks/week	1.20 (1.42)	1.25 (1.46)	0.54 (1.12)	0.63 (1.16)
Mean (SD) fish servings/week	0.32 (0.22)	0.31 (0.23)	0.32 (0.29)	0.31 (0.28)
Mean (SD) red meat servings/week ^2^	0.51 (0.31)	0.50 (0.32)	0.46 (0.40)	0.51 (0.42)
Mean (SD) fruit servings/week	3.01 (2.49)	2.86 (2.31)	3.70 (3.89)	3.64 (4.00)
Mean (SD) vegetable servings/week	0.80 (0.53)	0.79 (0.53)	0.98 (0.90)	1.02 (0.87)
Mean (SD) water, glasses/day ^2^	2.7 (2.2)	2.6 (2.2)	4.0 (2.6)	3.7 (2.4)
Mean (SD) coffee, cups/day ^2^	2.0 (2.0)	2.6 (2.3)	1.2 (1.6)	1.8 (2.1)
Coffee type ^2^				
Decaffeinated (any type)	60,959 (19.3)	1310 (18.2)	2505 (18.0)	59 (14.4)
Instant	176,056 (55.8)	3776 (52.5)	7922 (56.8)	198 (48.4)
Ground (includes espresso, filter etc.)	73,267 (23.2)	1980 (27.5)	2874 (20.6)	123 (30.1)
Other type	5335 (1.7)	129 (1.8)	644 (4.6)	29 (7.1)
Mean (SD) tea, cups/day ^2^	3.4 (2.7)	3.5 (2.9)	2.7 (2.3)	3.2 (2.5)
Non-tea/Non-regular coffee consumers	19,132 (4.8%)	347 (4.1%)	1437 (6.1)	10 (1.8)
Mean (SD) coffee/tea derived caffeine, mg/day ^2^	260 (157)	296 (179)	180 (139)	245 (177.5)
Mean (SD) fasting time, h^2^	3.8 (2.3)	3.1 (2.8)	4.5 (3.0)	3.4 (3.1)
Exam time, time ^2^	13:13 (2.8 h)	12:40 (2.8 h)	13:28 (2.8 h)	12:56 (2.6)
8 to 10 am	28,101 (20.5)	314 (26.1)	2428 (18.0)	40 (18.3)
11 am to 12 pm	30,804 (22.5)	266 (22.2)	2969 (22.0)	71 (32.4)
1–2 pm	29,405 (21.5)	282 (23.5)	2915 (21.6)	39 (17.8)
3–4 pm	28,146 (20.6)	225 (18.7)	2847 (21.1)	45 (20.6)
5 pm and later	20,402 (15.0)	114 (9.5)	2336 (17.3)	24 (11.0)
*APOE* ε4 carriers	83,495 (28.9)	1710 (28.1)	5776 (27.1)	116 (23.7)

^1^ Data drawn from 2006–2010 for participants with information on recent caffeine intake and who completed at least one of the cognitive function tests. Values are numbers (percentages) unless stated otherwise. For whites, all characteristic values are significantly different across recent caffeine drinking status (*p* < 0.05), with the exceptions of physical activity (*p* = 0.21), diabetes (*p* = 0.83), self-reported health (*p* = 0.06), vegetable intake (*p* = 0.08), and *APOE* (*p* = 0.21). ^2^ For non-white participants, significantly different between participants reporting recent and no recent caffeine drinking (*p* < 0.05). Abbreviations: CSE, certificate of secondary education; GCSE, general certificate of secondary education; NVQ, national vocational qualification; HND, higher national diploma; HNC, higher national certificate.

**Table 2:** Associations between recent caffeine drinking and cognitive function tests among white participants.

Recent Caffeine Drinking	*N*	Mean (SD) Score	Model 1 ^1^		Model 2 ^2^		Model 3 ^3^	
			β (95% CI)	*p*	β (95% CI)	*p*	β (95% CI)	*p*
^4^ Fluid Intelligence								
No	134,090	6.16 (2.11)	Reference		Reference		Reference	
Yes	1166	6.01 (2.12)	−0.20 (−0.32, −0.08)	0.001	−0.16 (−0.27, −0.05)	0.004	−0.16 (−0.27, −0.05)	0.004
^5^ Reaction Time								
No	398,870	553 (107)	Reference		Reference		Reference	
Yes	8472	540 (104)	−4.39 (−6.57, −2.21)	< 0.0001	−4.46 (−6.65, −2.28)	< 0.0001	−4.73 (−6.92, −2.54)	< 0.0001
^5^ Pairs Matching								
No	394,442	1.46 (0.62)	Reference		Reference		Reference	
Yes	8364	1.47 (0.63)	0.04 (0.02, 0.05)	< 0.0001	0.03 (0.02, 0.05)	< 0.0001	0.03 (0.02, 0.05)	< 0.0001
^4^ Prospective Memory								
	N	% correct	OR (95% CI)	*p*	OR (95% CI)	*p*	OR (95% CI)	*p*
No	136,708	79.8	Reference		Reference		Reference	
Yes	1200	76.2	0.73 (0.64, 0.83)	< 0.0001	0.78 (0.67, 0.90)	0.0005	0.78 (0.68, 0.90)	0.0006

^1^ Model 1: adjusted for age and sex. ^2^ Model 2: adjusted for age, sex, smoking, Townsend deprivation index, education, income, employment status, home-ownership, self-reported health, alcohol intake, water intake, fish intake, red meat intake, fruit intake, vegetable intake, waist-to-hip ratio, physical activity, and fasting time. ^3^ Model 3: Model 2 further adjusted for habitual coffee and tea intake. ^4^ Positive beta-coefficients for fluid intelligence and OR > 1 for PM correspond to higher performance compared to non-drinkers. ^5^ Negative beta-coefficients for pairs matching and reaction time correspond to higher performance compared to non-drinkers. Abbreviations: CI, confidence interval; OR, odds ratio.

**Table 3:** Associations between recent caffeine drinking and cognitive function tests among non-white participants.

Recent Caffeine Drinking	N	Mean (SD) Score	Model 1 ^1^		Model 2 ^2^		Model 3 ^3^	
			β (95% CI)	*p*	β (95% CI)	*p*	β (95% CI)	*p*
^4^ Fluid Intelligence								
No	11,430	4.34 (1.93)	Reference		Reference		Reference	
Yes	176	4.12 (1.84)	−0.27 (−0.55, −0.01)	0.06	−0.24 (−0.50,−0.02)	0.02	−0.22 (−0.48, 0.03)	0.09
^5^ Reaction Time								
No	23,079	595 (135)	Reference		Reference		Reference	
Yes	544	588 (134)	5.46 (−5.62, 16.54)	0.33	7.89 (−2.98, 18.75)	0.15	7.95 (−2.92, 18.82)	0.15
^5^ Pairs Matching								
No	21,558	1.65 (0.67)	Reference		Reference		Reference	
Yes	514	1.67 (0.68)	0.06 (0.003, 0.12)	0.04	0.07 (0.009, 0.12)	0.02	0.06 (0.007, 0.12)	0.03
^4^ Prospective Memory								
	*N*	% correct	OR (95% CI)	*p*	OR (95% CI)	*p*	OR (95% CI)	*p*
No	13,438	50.0	Reference		Reference		Reference	
Yes	218	46.8	0.83 (0.63, 1.08)	0.16	0.86 (0.64, 1.14)	0.29	0.87 (0.65, 1.16)	0.34

^1^ Model 1: adjusted for age, sex, and ethnicity. ^2^ Model 2: adjusted for age, sex, ethnicity, smoking, Townsend deprivation index, education, income, employment status, home-ownership, self-reported health, alcohol intake, fish intake, red meat intake, fruit intake, vegetable intake, waist-to-hip ratio, physical activity, and fasting time. ^3^ Model 3: Model 2 further adjusted for habitual coffee and tea intake. ^4^ Positive beta-coefficients for FI and OR > 1 for PM correspond to higher performance compared to non-consumers. ^5^ Negative beta-coefficients for Pairs and RT correspond to higher performance compared to non-consumers.

**Table 4:** Associations between recent caffeine drinking and cognitive function tests among white participants stratified by genetic caffeine metabolism score (CMS_G_) ^1^.

Recent Caffeine Drinking	CMS_G_ 0–1		CMS_G_ 1–2		CMS_G_ 3–4	
	β (95% CI)	*p*	β (95% CI)	*p*	β (95% CI)	*p*
^2^ Fluid Intelligence						
No	Reference		Reference		Reference	
Yes	−0.13 (−0.38, 0.12)	0.51	−0.07 (−0.26, 0.11)	0.43	−0.16 (−0.43, 0.11)	0.26
^3^ Reaction Time						
No	Reference		Reference		Reference	
Yes	−4.91 (−9.61, −0.21)	0.04	−5.24 (−9.08, −1.40)	0.01	−9.02 (−14.15, −3.89)	0.0006
^3^ Pairs Matching						
No	Reference		Reference		Reference	
Yes	0.02 (−0.01, 0.05)	0.18	0.03 (0.01, 0.05)	0.01	0.05 (0.01, 0.08)	0.004
^2^ Prospective Memory						
	OR (95% CI)	*p*	OR (95% CI)	*p*	OR (95% CI)	*p*
No	Reference		Reference		Reference	
Yes	0.75 (0.54, 1.04)	0.08	0.92 (0.71, 1.20)	0.55	0.67 (0.47, 0.95)	0.03

^1^ Results from Model 3: adjusted for age, sex, smoking, Townsend deprivation index, education, income, employment status, home-ownership, self-reported health, alcohol intake, water intake, fish intake, red meat intake, fruit intake, vegetable intake, waist-to-hip ratio, physical activity, fasting time, coffee intake, and tea intake. ^2^ Positive beta-coefficients for FI and OR > 1 for PM correspond to higher performance compared to non-drinkers. ^3^ Negative beta-coefficients for Pairs and RT correspond to higher performance compared to non-drinkers.

## Supplementary Materials

The following are available online at https://www.mdpi.com/2072-6643/12/7/1969/s1, Table S1: Assessment Center Order of Operations (Main Protocol (1)); Table S2: Baseline Characteristics of Unrelated (White) British Ancestry UK Biobank Participants According To Genetic Caffeine Metabolism Score (CMS_G_)*. Values Are Numbers (Percentages) Unless Stated Otherwise; Table S3: Associations between Recent Caffeine Drinking and Cognitive Function Tests among White Participants Stratified by Age (*p* > 0.05 for all interactions)*; Table S4: Associations between Recent Caffeine Drinking and Cognitive Function Tests among Non-White Participants Stratified by Age (*p* > 0.28 for all interactions)*; Table S5: Associations between Recent Caffeine Drinking and Cognitive Function Tests among White Participants Stratified by Exam time (*p* > 0.47 for all interactions)*; Table S6: Associations between Recent Caffeine Drinking and Cognitive Function Tests among Non-White Participants Stratified by Exam time (*p* > 0.09 for all interactions)*; Table S7: Associations Between Recent Caffeine Drinking and Cognitive Function Tests Among White Participants Stratified by Habitual Caffeine Intake*; Table S8: Associations between Recent Caffeine Drinking and Cognitive Function Tests among Non-White Participants Stratified by Habitual Caffeine Intake (*p* > 0.16 for all interactions)*; Table S9: Associations between Recent Caffeine Drinking and Cognitive Function Tests among White Participants Stratified by APOE ε4 Carrier Status*; Table S10: Associations Between Recent Caffeine Drinking and Cognitive Function Tests Among Non-White Participants Stratified by Smoking Status*; Table S11: Associations Between Recent Caffeine Drinking and Cognitive Function Tests Among White Participants Stratified by Habitual Caffeine Intake and Exam Time*; Table S12: Associations Between Recent Caffeine Drinking and Cognitive Function Tests Among White Participants Stratified by Age and Exam Time*; Table S13: Associations between Recent Caffeine Drinking and Cognitive Function Tests among White Participants Stratified by rs6968554 genotype (*p* > 0.05 for all interactions)*; Table S14: Associations between Recent Caffeine Drinking and Cognitive Function Tests among White Participants Stratified by rs2472297 genotype (*p* > 0.05 for all interactions)*; Table S15: Associations between Recent Caffeine Drinking and Cognitive Function Tests among White Participants Stratified by rs762551 (*p* > 0.05 for all interactions)*.

## Abbreviations

CMS	Caffeine-metabolism Score
FI	fluid intelligence
Pairs	pairs matching
PM	prospective memory
RT	reaction time
SNP	single-nucleotide polymorphism
WHR	waist-to-hip ratio
