# Supplementary material for: Recent Caffeine Drinking Associates with Cognitive Function in the UK Biobank
Source: Nutrients. 2020 Jul 2;12(7):1969. doi: 10.3390/nu12071969 (PMC7399821; doi:10.3390/nu12071969)
Supplement: Supplementary file 1 [file nutrients-12-01969-s001.pdf]

Recent Caffeine Drinking Associates with Cognitive Function in the UK Biobank

Supplementary Data

Supplementary Method

Participants

UK Biobank investigators sent postal invitations to approximately 9.2 million individuals registered with the UK's National Health Service who were aged 40–69 years and lived within approximately 40 km of one of 22 assessment centers located throughout England, Wales, and Scotland [1]. About 3 weeks after mailing the invitation letter, people who did not respond were sent a re-invitation letter once only. Personal invitations were paralleled with increasing local awareness of the UK Biobank [1]. Between 2006 and 2010, over 502,633 participants aged 37–73 years (5.4% participation rate) provided full informed consent to participate in the UK Biobank and completed a 90-minute assessment that included, (i) touchscreen questionnaires on sociodemographic factors, lifestyle, and medical history, (ii) an in-person interview, and (iii) physical assessment: (see Table S1 for order of operations). Usual assessment centre opening hours were Monday to Friday 8:00 am to 8:00 pm (last appointment starting at 7:00 pm) and Saturday 8:00 am to 6:00 pm (last appointment starting at 5:00 pm).

Measures and Procedures

The UK Biobank baseline questions and measurements have been chosen carefully to allow a wide assessment of health-related outcomes to be conducted in the whole cohort. The questionnaire was administered in two sequential parts during the assessment center visit: a touchscreen self-completed questionnaire followed by a computer-assisted personal interview (CAPI). Due to the large size of the

UK Biobank cohort, the approach to data capture aimed to optimize the accuracy and completeness of the data collected, while also maximizing the efficiency of the process. A pre-visit aide memoire is provided to participants prior to attending the assessment center so that they can note certain information (e.g., medications, operations, family history, and birth details) that may be difficult or time-consuming for them to recall during the visit. Pre-coded lists of diseases, drugs, and occupations are built into the CAPI system, along with structured search facilities, to help this information to be recorded (and automatically coded) both rapidly and completely. Data for the current analysis was downloaded in 2017.

Cognitive function testing

Cognitive functioning [2] was assessed using a 15-min computerized battery which was developed specifically for the UK Biobank study to enable population-scale cognitive testing that could be administered without researcher supervision. Following wide consultation, a comprehensive review was conducted of brief tests of cognition that can be self-administered, are easily repeatable within a larger cognitive screening battery [3], and have associations with future cognitive decline. Based on this review, paired associated learning questions to assess global cognition [4] and reaction time tests for touchscreen administration have been developed and refined through piloting to ensure that they provide wide response distributions [1]. A detailed description of all tests are provided online [5,6] and summarized in Table S1 and sections below.

Prospective Memory (PM) Test: This test was added part-way through the baseline assessment period. Participants were given the following instructions: “At the end of the games, we will show

you four colored symbols and ask you to touch the blue square. However, to test your memory, we want you to actually touch the orange circle instead." Participants were scored as zero or one, depending on whether they completed the task on the first attempt or not. This test assesses PM: the ability to carry out future intentions at a specific time or in response to a specific event, and therefore includes a prospective component (remembering to remember) and a retrospective component (remembering the content of what is to be remembered) [7,8].

Pairs Matching (Pairs) Test: This episodic visual memory test was completed at the assessment centers. Participants were shown 6 pairs of cards for 5 seconds, which were then turned over. Participants were instructed to select, from recall and in the fewest number of attempts, the pairs of cards that had matching symbols. There was no time limit and the participants could make as many attempts as they needed to find all the pairs. The memory test score in the present study is the total number of errors made during this task until the six pairs of identical cards were touched consecutively. We restricted our analyses to individuals who finished the test and log (+1) transformed the number of errors for the analysis. During the pilot phase of the UK Biobank, a subset of participants completed this test twice in immediate succession and the intraclass correlation was 0.17 [9].

Fluid Intelligence (FI) Test: This test was added part-way through the baseline assessment period. Participants were presented with 13 verbal logic/reasoning-type multiple choice questions and had to answer as many as they could within 2 minutes. There were six verbal items and seven numerical items, involving sequence recognition and arithmetic. Incorrect or unattempted questions were scored as zero. The total number of correct answers (max 13) was used for our analysis. The

Cronbach alpha coefficient for these items has been reported elsewhere as 0.62 [10]. The UK Biobank describes FI as the "capacity to solve problems that require logic and reasoning ability, independent of acquired knowledge" [5]. Others have preferred the test label 'verbal-numerical reasoning' as opposed to the UK Biobank's label 'fluid Intelligence' since performance on some items more likely rely on crystalized knowledge [9–13].

Reaction Time (RT) Test: For this measure of simple processing speed, participants completed a timed test of symbol matching at the assessment centers. Participants were shown one pair of cards out of a set of 12 pairs. If both cards displayed a matching symbol, participants pressed a response button as quickly as possible using their dominant hand. Five 'training' trials were administered, followed by seven test trials. The score for analysis was the mean time (in milliseconds) to press the button, derived from the four trials in which a matching pair occurred. The UK Biobank indicates that, (i) times under 50 ms must be due to anticipation rather than reaction and so were excluded, and (ii) times over 2000 ms are ignored since cards had disappeared by then. For the current analysis, additional potential outliers were truncated (not excluded) to 100 (min) or 1000 (max) ms as they scored more than 4 SD away from the mean. This approach generated better normally distributed scores than log-transformed scores. Cronbach's alpha for this task has previously been reported as 0.85 [10].

Table S1. Assessment Center Order of Operations (Main Protocol (1))

				Current analysis		
**Visit station**	**Assessments undertaken**	**Variables for current analysis**	Data available	White Participants	Non-White Participants	Subset with genetic data†
**Reception (10 min)**	**Welcome and registration<br>Consent**					

		Age				
		Sex				
		Race				
		Smoking status				
		Income				
		Education				
		Employment	502,616	410,183	24,717	295,492
		Self-rate health	502,616	410,183	24,717	295,492
		Townsend	502,616	410,183	24,717	295,492
		Physical activity	502,616	410,183	24,717	295,492
		Coffee	425,419	410,183	24,717	295,492
		Coffee type	407,183	410,183	24,717	295,492
	Touchscreen	Tea	496,857	410,183	24,717	295,492
	questionnaire	Alcohol	499,125	408,809	24,221	294,610
		Water	501,989	409,681	24,669	295,133
		Fish	491,884	401,223	24,252	288,993
		Red meat	500,347	409,568	24,305	295,063
		Fruit	384,485	322,828	14,354	233,578
		Vegetables	500,421	409,450	24,492	294,998
		**Cognitive**	502,616	410,183	24,717	295,492
		**function testing**	497,833	407,544	24,218	293,658
Questionnaire		1: Prospective	500,561	409,696	24,339	295,182
(40 min)		Memory:	501,356	410,076	24,582	295,416
		Shape—Part 1	500,474	409,626	24,377	295,118
		Test	498,126	408,060	24,078	294,036
		initiation time				
		2: Pairs	171,309	137,908	13,656	98,461
		matching test				
		3: Fluid	171,577	138,059	13,714	98,559
		intelligence	482,735	402,806	22,072	290,580
		4: Reaction	165,486	135,256	11,606	96,753
		time—Snap	490,516	407,342	23,623	293,610
		1: Prospective	171,309	137,908	13,656	98,461
		Memory:				
		Shape—Part 2	502,616	410,183	24,717	295,492
		Self-reported				
		diabetes, insulin	502,616	410,183	24,717	295,492
		use, and/or diabetic medicine				
		Self-reported blood pressure medication				
	Interviewer	Medical conditions*	501,674	410,183	24,717	295,492
Interview	questionnaire					
(10 min)	Blood pressure	SBP	501,290	409,858	24,691	295,271
	measurement	DBP	501,292	409,860	24,691	295,273

Physical measurements (15 min)	Anthropometrics, hand-grip strength, heel bone ultrasound, spirometry	BMI	499,511	409,567	24,536	295,058
		WHR	500,351	409,953	24,697	295,339
		Caffeine consumed in last hour (y/n)	460,110	410,183	24,717	295,492
Sample collection (15 min)	Blood sample	Fasting time	501,383	410,011	24,702	295,480
	Urine sample	QC Genetic data	407,206	334,078		295,492
Exit	Consent and result summary printed<br>Travel expense claim provided					

*Interview questionnaire on medical conditions was used for identifying participants with neurological conditions.

†The subset of participants for genetic analysis included unrelated individuals with high-quality genetic data, who self-report as "British" and who have very similar ancestral backgrounds based on results of principal component analysis (see Methods in main paper for details)

Table S2. Baseline Characteristics of Unrelated (White) British Ancestry UK Biobank Participants According To Genetic Caffeine Metabolism Score (CMS$_G$)*. Values Are Numbers (Percentages) Unless Stated Otherwise

**Characteristic**	**CMS$_G$ 0–1 N = 94,876**	**CMS$_G$ 1–2 N = 132,787**	**CMS$_G$ 3–4 N = 67,829**
Mean (SD) age, years	56.6 (8.0)	56.6 (8.0)	56.6 (8.0)
Male	43,183 (46.0)	60,566 (45.6)	33,818 (46.0)
Smoking status			
Never	52,371 (55.2)	73,792 (55.6)	37,641 (55.5)
Past	32,990 (34.8)	45,850 (34.5)	23,510 (34.7)
Current	9515 (10.0)	13,145 (9.9)	6678 (9.9)
Mean (SD) BMI, kg/m^2†	27.2 (4.7)	27.3 (4.7)	27.4 (4.7)
Mean (SD) waist-to-hip ratio†	0.87 (0.09)	0.87 (0.09)	0.87 (0.09)
Income			
<18,000	16,648 (17.6)	23,708 (17.9)	12,186 (18.0)
18,000–30,999	21,151 (22.3)	29,303 (22.1)	14,927 (22.0)
31,000–51,999	22,138 (23.3)	30,988 (23.3)	15,724 (23.2)
52,000–100,000	17,648 (18.6)	24,569 (18.5)	12,585 (18.6)
'100,000+	4557 (4.8)	6503 (4.9)	3244 (4.8)
will not answer, missing	12,734 (13.4)	17,716 (13.3)	9163 (13.5)
Education			
None (listed), prefer not to answer	15,979 (16.8)	22,528 (17.0)	11,594 (17.1)
CSEs or equivalent	3567 (3.8)	4977 (3.8)	2503 (3.7)
O levels/GCSEs or equivalent	12,814 (13.5)	17,850 (13.4)	9334 (13.8)
A levels/AS levels or equivalent	5079 (5.4)	7366 (5.6)	3646 (5.4)
Other professional qualifications	11,845 (12.5)	16,456 (12.4)	8268 (12.2)
NVQ or HND or HNC or equivalent	14,822 (15.6)	20,957 (15.8)	10,745 (15.8)
College or University degree	30,770 (32.4)	42,653 (32.1)	21,739 (32.0)

Employment status			
currently employed	55,453 (58.5)	77,570 (58.4)	39,503 (58.2)
retired	32,261 (34.0)	45,186 (34.0)	23,084 (34.0)
other/not reported	7162 (7.6)	10,031 (7.6)	5242 (7.7)
Mean (SD) Townsend deprivation score	–1.63 (2.89)	–1.64 (2.89)	–1.62 (2.90)
Home owner	86,313 (91.0)	120,892 (91.0)	61,618 (90.8)
Mean (SD) moderate to vigorous physical activity, minutes/week	77 (97)	77 (96)	77 (97)
Hypertension	50,221 (52.9)	70,588 (53.2)	35,895 (52.9)
Diabetes	4095 (4.3)	5949 (4.5)	3057 (4.5)
Self-reported overall health rating†			
Excellent	17,056 (18.0)	23,730 (17.9)	11,880 (17.6)
Good	56,780 (60.0)	79,446 (60.0)	40,516 (59.9)
Fair	17,765 (18.8)	25,112 (19.0)	13,018 (19.3)
Poor	2992 (3.2)	4096 (3.1)	2219 (3.3)
Mean (SD) alcohol drinks/week†	1.20 (1.42)	1.21 (1.41)	1.23 (1.45)
Mean (SD) fish servings/week	0.32 (0.22)	0.32 (0.22)	0.32 (0.22)
Mean (SD) red meat servings/week†	0.51 (0.31)	0.51 (0.31)	0.51 (0.31)
Mean (SD) fruit servings/week†	3.01 (2.50)	2.99 (2.44)	2.93 (2.40)
Mean (SD) vegetable servings/week	0.79 (0.51)	0.79 (0.51)	0.79 (0.51)
Mean (SD) water, glasses/d†	2.7 (2.2)	2.6 (2.2)	2.5 (2.1)
Mean (SD) coffee, cups/d†	1.9 (1.9)	2.1 (2.0)	2.2 (2.1)
Coffee type†			
Decaffeinated (any type)	14,758 (20.0)	20,639 (19.6)	10,587 (19.4)
Instant	41,867 (56.8)	59,030 (56.1)	30,184 (55.3)
Ground (includes espresso, filter etc.)	15,899 (21.6)	23,788 (22.6)	12,981 (23.8)
Other type	1223 (1.7)	1743 (1.7)	879 (1.6)
Mean (SD) tea, cups/d†	3.3 (2.6)	3.4 (2.7)	3.6 (2.8)
Mean (SD) coffee/tea derived caffeine, mg/d†	246 (150)	263 (157)	280 (164)
Mean (SD) fasting time, hours†	3.7 (2.3)	3.7 (2.3)	3.7 (2.3)
Mean (SD) exam time, time	13:11 (2.8)	13:12 (2.8)	13:20 (2.8)
Recent caffeine drinking†	1813 (1.9)	2746 (2.1)	1523 (2.3)
APOE ε4 carriers	27,483 (29.0)	38,358 (28.9)	19,364 (28.6)

*Data drawn from 2006–2010 for participants with information on recent caffeine intake, genome-wide data, and who completed at least one of the cognitive function tests.
†Significantly different across CMS_G ($p < 0.05$).

Table S3. Associations Between Recent Caffeine Drinking and Cognitive Function Tests Among White Participants Stratified by Age *(p* > 0.05 for all interactions)*

Recent Caffeine	<55 years of age β (95% CI)	*p*	55+ years of age β (95% CI)	*p*
**§Fluid Intelligence**				
No	Reference		Reference	
Yes	–0.15 (–0.31, 0.01)	0.06	–0.17 (–0.32, –0.1)	0.03
**¶Reaction Time**				
No	Reference		Reference	
Yes	–4.82 (–7.63, –2.01)	0.0008	–4.61 (–7.84, –1.38)	0.01
**¶Pairs Matching**				
No	Reference		Reference	
Yes	0.03 (0.01, 0.05)	0.002	0.04 (0.02, 0.05)	0.0002
**§Prospective Memory Test**				
	**OR (95% CI)**	*p*	**OR (95% CI)**	*p*
No	Reference		Reference	
Yes	0.79 (0.64, 0.99)	0.04	0.77 (0.64, 0.93)	0.01

*Results from Model 3: adjusted for age, sex, smoking, Townsend deprivation index, education, income, employment status, home-ownership, self-reported health, alcohol intake, water intake, fish intake, red meat intake, fruit intake, vegetable intake, waist-to-hip ratio, physical activity, fasting time, coffee intake, and tea intake.

§Positive beta-coefficients for FI and OR >1 for PM correspond to higher performance compared to non-consumers.

¶Negative beta-coefficients for Pairs and RT correspond to higher performance compared to non-consumers.

Table S4. Associations Between Recent Caffeine Drinking and Cognitive Function Tests Among Non-White Participants Stratified by Age *(p* > 0.28 for all interactions)*

Recent Caffeine	<55 years of age β (95% CI)	*p*	55+ years of age β (95% CI)	*p*
**§Fluid Intelligence**				
No	Reference		Reference	
Yes	–0.18 (–0.51, 0.14)	0.27	–0.25 (–0.67, 0.17)	0.25
**¶Reaction Time**				
No	Reference		Reference	
Yes	8.46 (–4.10, 21.01)	0.19	3.08 (–17.66, 23.82)	0.77
**¶Pairs Matching**				
No	Reference		Reference	
Yes	0.08 (0.01, 0.15)	0.02	0.01 (–0.09, 0.11)	0.83
**§Prospective Memory Test**				
	**OR (95% CI)**	*p*	**OR (95% CI)**	*p*
No	Reference		Reference	
Yes	0.98 (0.68, 1.41)	0.91	0.77 (0.48, 1.25)	0.29

*Results from Model 3: adjusted for age, sex, race, smoking, Townsend deprivation index, education, income, employment status, home-ownership, self-reported health, alcohol intake, water intake, fish intake, red meat intake, fruit intake, vegetable intake, waist-to-hip ratio, physical activity, fasting time, coffee intake, and tea intake.

§Positive beta-coefficients for FI and OR >1 for PM correspond to higher performance compared to non-consumers.

¶Negative beta-coefficients for Pairs and RT correspond to higher performance compared to non-consumers.

Table S5. Associations Between Recent Caffeine Drinking and Cognitive Function Tests Among White Participants Stratified by Exam time ($p > 0.47$ for all interactions)*

	≤12 pm		>12 pm	
**Recent Caffeine**	**β (95% CI)**	***p***	**β (95% CI)**	***p***
	**§Fluid Intelligence**			
No	Reference		Reference	
Yes	−0.14 (−0.30, 0.02)	0.08	−0.18 (−0.33, −0.02)	0.02
	**¶Reaction Time**			
No	Reference		Reference	
Yes	−1.21 (−9.87, 7.44)	0.78	1.99 (−6.63, 10.61)	0.65
	**¶Pairs Matching**			
No	Reference		Reference	
Yes	0.01 (−0.04, 0.07)	0.61	−0.005(−0.05, 0.05)	0.86
	**§Prospective Memory**			
	**OR (95% CI)**	*p*	**OR (95% CI)**	*p*
No	Reference		Reference	
Yes	0.80 (0.65, 0.99)	0.04	0.77 (0.64, 0.94)	0.01

*Results from Model 3: adjusted for age, sex, smoking, Townsend deprivation index, education, income, employment status, home-ownership, self-reported health, alcohol intake, water intake, fish intake, red meat intake, fruit intake, vegetable intake, waist-to-hip ratio, physical activity, fasting time, coffee intake, and tea intake. Similar results were observed when using different cut-points for exam-time.

§Positive beta-coefficients for FI and OR >1 for PM correspond to higher performance compared to non-consumers.

¶Negative beta-coefficients for Pairs and RT correspond to higher performance compared to non-consumers.

Table 6. Associations Between Recent Caffeine Drinking and Cognitive Function Tests Among Non-White Participants Stratified by Exam time ($p > 0.09$ for all interactions)*.

	≤12 pm		>12 pm	
**Recent Caffeine**	**β (95% CI)**	***p***	**β (95% CI)**	***p***
	**§Fluid Intelligence**			
No	Reference		Reference	
Yes	−0.19 (−0.56, 0.18)	0.32	−0.27 (−0.63, 0.10)	0.15
	**¶Reaction Time**			
No	Reference		Reference	
Yes	37.69 (13.29, 62.10)	0.003	−0.04 (−26.01, 25.93)	0.99
	**¶Pairs Matching**			
No	Reference		Reference	
Yes	0.03 (−0.10, 0.16)	0.66	0.18 (0.05, 0.32)	0.01
	**§Prospective Memory**			
	**OR (95% CI)**	*p*	**OR (95% CI)**	*p*
No	Reference		Reference	
Yes	0.77 (0.51, 1.15)	0.20	0.97 (0.64, 1.47)	0.89

*Results from Model 3: adjusted for age, sex, race, smoking, Townsend deprivation index, education, income, employment status, home-ownership, self-reported health, alcohol intake, water intake, fish intake, red meat intake, fruit intake, vegetable intake, waist-to-hip ratio, physical activity, fasting time, coffee intake, and tea intake.
§Positive beta-coefficients for FI and OR >1 for PM correspond to higher performance compared to non-consumers.
¶Negative beta-coefficients for Pairs and RT correspond to higher performance compared to non-consumers.

Table S7. Associations Between Recent Caffeine Drinking and Cognitive Function Tests Among White Participants Stratified by Habitual Caffeine Intake*

	**≤100 mg/d**		**100+ mg/d**	
**Recent Caffeine**	**β (95% CI)**	***p***	**β (95% CI)**	***p***
	**§Fluid Intelligence**			
No	Reference		Reference	
Yes	–0.02 (–0.35, 0.32)	0.92	–0.18 (–0.30, –0.06)	0.003
	**¶Reaction Time**			
No	Reference		Reference	
Yes	–4.91 (–11.56, 1.73)	0.15	–4.57 (–6.89, –2.25)	0.0001
	**¶Pairs Matching**			
No	Reference		Reference	
Yes	0.05 (0.01, 0.09)	0.03	0.03 (0.02, 0.05)	<.0001
	**†§Prospective Memory**			
	**OR (95% CI)**	***p***	**OR (95% CI)**	***p***
No	Reference		Reference	
Yes	0.53 (0.36, 0.79)	0.002	0.81 (0.70, 0.94)	0.01

*Results from Model 3: adjusted for age, sex, smoking, Townsend deprivation index, education, income, employment status, home-ownership, self-reported health, alcohol intake, water intake, fish intake, red meat intake, fruit intake, vegetable intake, waist-to-hip ratio, physical activity, fasting time, coffee intake, and tea intake.
† $p = 0.02$ for habitual caffeine intake × recent caffeine interaction for PM. All other tests for interaction $p > 0.31$.
§Positive beta-coefficients for FI and OR > 1 for PM correspond to higher performance compared to non-consumers.
¶Negative beta-coefficients for Pairs and RT correspond to higher performance compared to non-consumers.

Table S8. Associations Between Recent Caffeine Drinking and Cognitive Function Tests Among Non-White Participants Stratified by Habitual Caffeine Intake ($p \geq 0.16$ for all interactions)*

	**≤100 mg/d**		**100+ mg/d**	
**Recent Caffeine**	**β (95% CI)**	***p***	**β (95% CI)**	***p***
	**§Fluid Intelligence**			
No	Reference		Reference	
Yes	0.17 (–0.49, 0.82)	0.62	–0.32 (—0.60, –0.03)	0.03
	**¶Reaction Time**			
No	Reference		Reference	
Yes	–5.78 (–34.96, 23.41)	0.70	9.37 (–2.7, 21.01)	0.11
	**¶Pairs Matching**			
No	Reference		Reference	
Yes	0.16 (0.01, 0.31)	0.03	0.05 (–0.02, 0.11)	0.14

Supplementary Data

	§Prospective Memory			
	**OR (95% CI)**	***p***	**OR (95% CI)**	***p***
No	Reference		Reference	
Yes	1.35 (0.65, 2.79)	0.42	0.78 (0.57, 1.07)	0.12

*Results from Model 3: adjusted for age, race, sex, smoking, Townsend deprivation index, education, income, employment status, home-ownership, self-reported health, alcohol intake, water intake, fish intake, red meat intake, fruit intake, vegetable intake, waist-to-hip ratio, physical activity, fasting time, coffee intake, and tea intake.§Positive beta-coefficients for FI and OR > 1 for PM correspond to higher performance compared to non-consumers.¶Negative beta-coefficients for Pairs and RT correspond to higher performance compared to non-consumers.

References

1. UK Biobank Coordinating Centre. UK Biobank: Protocol for a large-scale prospective epidemiological resource; 2007.
2. Cullen B, Nicholl BI, Mackay DF, Martin D, Ul-Haq Z, McIntosh A, Gallacher J, Deary IJ, Pell JP, et al. Cognitive function and lifetime features of depression and bipolar disorder in a large population sample: Cross-sectional study of 143,828 UK Biobank participants. Eur Psychiatry. 2015 Nov;30:950-8.
3. Brayne C, Day N, Gill C. Methodological issues in screening for dementia. Neuroepidemiology. 1992;11:88-93.
4. De Jager C, Blackwell AD, Budge MM, Sahakian BJ. Predicting cognitive decline in healthy older adults. The American Journal of Geriatric Psychiatry. 2005;13:735-40.
5. UK Biobank Coordinating Centre. Cognitive Function Tests: Assessment Centre. 2006 [cited 2018 October]; Available from: http://biobank.ctsu.ox.ac.uk/crystal/label.cgi?id=100026
6. UK Biobank Coordinating Centre. Cognitive Function Tests: On-line. 2014 [cited 2018 October]; Available from: http://biobank.ctsu.ox.ac.uk/crystal/label.cgi?id=116
7. McDaniel MA, Scullin MK. Implementation intention encoding does not automatize prospective memory responding. Memory & Cognition. 2010;38:221-32.
8. Cleutjens FA, Spruit MA, Ponds RW, Dijkstra JB, Franssen FM, Wouters EF, Janssen DJ. Cognitive functioning in obstructive lung disease: results from the United Kingdom biobank. Journal of the American Medical Directors Association. 2014;15:214-9.
9. Lyall DM, Cullen B, Allerhand M, Smith DJ, Mackay D, Evans J, Anderson J, Fawns-Ritchie C, McIntosh AM, Deary IJ. Cognitive test scores in UK Biobank: data reduction in 480,416 participants and longitudinal stability in 20,346 participants. PloS one. 2016;11:e0154222.
10. Hagenaars SP, Harris SE, Davies G, Hill WD, Liewald DC, Ritchie SJ, Marioni RE, Fawns-Ritchie C, Cullen B, et al. Shared genetic aetiology between cognitive functions and physical and mental health in UK Biobank (N=112 151) and 24 GWAS consortia. Molecular psychiatry. 2016 Nov;21:1624-32.
11. Davies G, Lam M, Harris SE, Trampush JW, Luciano M, Hill WD, Hagenaars SP, Ritchie SJ, Marioni RE, Fawns-Ritchie C. Study of 300,486 individuals identifies 148 independent genetic loci influencing general cognitive function. Nature communications. 2018;9:2098.

12. Hill WD, Davies G, Harris S, Hagenaars S, Davies G, Deary IJ, Debette S, Verbaas CI, Bressler J, Schuur M. Molecular genetic aetiology of general cognitive function is enriched in evolutionarily conserved regions. Translational psychiatry. 2016;6:e980.
13. Nevado-Holgado AJ, Kim C-H, Winchester L, Gallacher J, Lovestone S. Commonly prescribed drugs associate with cognitive function: a cross-sectional study in UK Biobank. BMJ open. 2016;6:e012177.

Table S9. Associations Between Recent Caffeine Drinking and Cognitive Function Tests Among White Participants Stratified by *APOE* ε4 Carrier Status*

	***APOE* ε4 non-carriers**		***APOE* ε4 carriers**	
**Recent Caffeine**	**β (95% CI)**	***p***	**β (95% CI)**	***p***
**§Fluid Intelligence**				
No	Reference		Reference	
Yes	–0.07 (–0.23, 0.08)	0.36	–0.21 (–0.44, 0.03)	0.09
**¶Reaction Time**				
No	Reference		Reference	
Yes	–5.42 (–8.46, –2.38)	0.0005	–7.79 (–12.63, –2.96)	0.002
**†¶Pairs Matching**				
No	Reference		Reference	
Yes	0.04 (0.02, 0.06)	<0.0001	0.003 (–0.03, 0.03)	0.83
**§Prospective Memory**				
	**OR (95% CI)**	***p***	**OR (95% CI)**	***p***
No	Reference		Reference	
Yes	0.74 (0.60, 0.91)	0.004	0.99 (0.71, 1.39)	0.95

*Results from Model 3: adjusted for age, sex, smoking, Townsend deprivation index, education, income, employment status, home-ownership, self-reported health, alcohol intake, water intake, fish intake, red meat intake, fruit intake, vegetable intake, waist-to-hip ratio, physical activity, fasting time, coffee intake, and tea intake.

†p = 0.04 for *APOE* × recent caffeine interaction for Pairs. All other tests for interaction $p > 0.09$.

§Positive beta-coefficients for FI and OR > 1 for PM correspond to higher performance compared to non-consumers.

¶Negative beta-coefficients for Pairs and RT correspond to higher performance compared to non-consumers.

Table S10. Associations Between Recent Caffeine Drinking and Cognitive Function Tests Among Non-White Participants Stratified by Smoking Status*

	**Never smokers**		**Past smokers**		**Current smokers**	
**Recent Caffeine**	**β (95% CI)**	***p***	**β (95% CI)**	***p***	**β (95% CI)**	***p***
**§Fluid Intelligence**						
No	Reference		Reference		Reference	
Yes	–0.26 (–0.60, 0.08)	0.14	–0.38 (–0.99, 0.23)	0.23	0.01 (–0.52, 0.55)	0.96
**†¶Reaction Time**						
No	Reference		Reference		Reference	
Yes	–5.59 (–20.54, 9.35)	0.46	20.68 (–0.48, 41.84)	0.06	27.53 (4.45, 50.61)	0.02
**¶Pairs Matching**						
No	Reference		Reference		Reference	
Yes	0.07 (–0.002, 0.15)	0.06	0.10 (–0.02, 0.22)	0.10	–0.02 (–0.15, 0.11)	0.76
**§Prospective Memory**						
	**OR (95% CI)**	***p***	**OR (95% CI)**	***p***	**OR (95% CI)**	***p***
No	Reference		Reference		Reference	
Yes	0.87 (0.59, 1.29)	0.48	0.71 (0.36, 1.39)	0.32	0.90 (0.50, 1.61)	0.71

*Results from Model 3: adjusted for age, race, sex, smoking, Townsend deprivation index, education, income, employment status, home-ownership, self-reported health, alcohol intake, water intake, fish intake, red meat intake, fruit intake, vegetable intake, waist-to-hip ratio, physical activity, fasting time, coffee intake, and tea intake.

†$p = 0.007$ for smoking (3 categories) × recent caffeine interaction for RT ($p = 0.06$ when smoking modeled as nonsmokers versus smokers). All other tests for interaction $p > 0.35$.

§Positive beta-coefficients for FI and OR > 1 for PM correspond to higher performance compared to non-consumers.

¶Negative beta-coefficients for Pairs and RT correspond to higher performance compared to non-consumers.

Table S11. Associations Between Recent Caffeine Drinking and Cognitive Function Tests Among White Participants Stratified by Habitual Caffeine Intake and Exam Time*

Recent Caffeine	≤12 pm				>12 pm			
	≤100 mg/d		100+ mg/d		≤100 mg/d		100+ mg/d	
	**β (95% CI)**	***p***	**β (95% CI)**	***p***	**β (95% CI)**	***p***	**β (95% CI)**	***p***
**§Fluid Intelligence**								
No	Reference		Reference		Reference		Reference	
Yes	–0.09 (–0.57, 0.39)	0.72	–0.14 (–0.31, 0.02)	0.09	0.04 (–0.43, 0.51)	0.86	–0.21 (–0.38, –0.05)	0.01
**¶Reaction Time**								
No	Reference		Reference		Reference		Reference	
Yes	–6.33 (–31.94, 19.27)	0.63	–0.55 (–9.77, 8.66)	0.91	–8.70(–34.90, 17.49)	0.51	3.30 (–5.84, 12.44)	0.48
**¶Pairs Matching**								
No	Reference		Reference		Reference		Reference	
Yes	0.0001 (–0.15, 0.15)	0.99	0.01 (–0.04, 0.07)	0.63	–0.10 (–0.26, 0.05)	0.20	0.01 (–0.04, 0.06)	0.73
**§Prospective Memory Test**								
	**OR (95% CI)**	***p***	**OR (95% CI)**	***p***	**OR (95% CI)**	***p***	**OR (95% CI)**	***p***
No	Reference		Reference		Reference		Reference	
Yes	0.44 (0.25, 0.78)	0.05	0.87 (0.69, 1.09)	0.23	0.66 (0.37, 1.17)	0.15	0.77 (0.62, 0.94)	0.01

*Results from Model 3: adjusted for age, sex, smoking, Townsend deprivation index, education, income, employment status, home-ownership, self-reported health, alcohol intake, water intake, fish intake, red meat intake, fruit intake, vegetable intake, waist-to-hip ratio, physical activity, fasting time, coffee intake, and tea intake.

§Positive beta-coefficients for FI and OR > 1 for PM correspond to higher performance compared to non-consumers.

¶Negative beta-coefficients for Pairs and RT correspond to higher performance compared to non-consumers.

Table S12. Associations Between Recent Caffeine Drinking and Cognitive Function Tests Among White Participants Stratified by Age and Exam Time*

**Recent Caffeine**	**≤12 pm**				**>12 pm**			
	**<55 years of age**		**55+ years of age**		**<55 years of age**		**55+ years of age**	
	**β (95% CI)**	***p***	**β (95% CI)**	***p***	**β (95% CI)**	***p***	**β (95% CI)**	***p***
**§Fluid Intelligence**								
No	Reference		Reference		Reference		Reference	
Yes	–0.15 (–0.37, 0.08)	0.20	–0.15 (–0.37, 0.08)	0.20	–0.16 (–0.38, 0.06)	0.16	–0.18 (–0.39, 0.03)	0.09
**¶Reaction Time**								
No	Reference		Reference		Reference		Reference	
Yes	–4.61 (–15.34, 6.12)	0.40	2.02 (–11.18, 15.22)	0.76	–2.66 (–13.80, 8.48)	0.64	6.55 (–6.08, 19.19)	0.31
**¶Pairs Matching**								
No	Reference		Reference		Reference		Reference	
Yes	0.02 (–0.05, 0.09)	0.62	0.01 (–0.06, 0.08)	0.80	–0.03 (–0.10, 0.04)	0.43	0.02 (–0.05, 0.09)	0.59
**§Prospective Memory**								
	**OR (95% CI)**	***p***	**OR (95% CI)**	***p***	**OR (95% CI)**	***p***	**OR (95% CI)**	***p***
No	Reference		Reference		Reference		Reference	
Yes	0.81 (0.59, 1.11)	0.19	0.78 (0.59, 1.03)	0.08	0.80 (0.59, 1.08)	0.14	0.77 (0.59, 0.99)	0.04

*Results from Model 3: adjusted for age, sex, smoking, Townsend deprivation index, education, income, employment status, home-ownership, self-reported health, alcohol intake, water intake, fish intake, red meat intake, fruit intake, vegetable intake, waist-to-hip ratio, physical activity, fasting time, coffee intake, and tea intake.

§Positive beta-coefficients for FI and OR > 1 for PM correspond to higher performance compared to non-consumers.

¶Negative beta-coefficients for Pairs and RT correspond to higher performance compared to non-consumers.

Table S13. Associations Between Recent Caffeine Drinking and Cognitive Function Tests Among White Participants Stratified by rs6968554 genotype ($p > 0.05$ for all interactions)*

	AA		AG		GG	
**Recent Caffeine**	**β (95% CI)**	*p*	**β (95% CI)**	*p*	**β (95% CI)**	*p*
**§Fluid Intelligence**						
No	Reference		Reference		Reference	
Yes	–0.12 (–0.48, 0.24)	0.52	–0.12 (–0.32, 0.08)	0.23	–0.11 (–0.30, 0.09)	0.29
**¶Reaction Time**						
No	Reference		Reference		Reference	
Yes	–1.20 (–8.55, 6.16)	0.75	–6.08 (–9.90, –2.26)	0.002	–7.66 (–11.61, –3.71)	0.0001
**¶Pairs Matching**						
No	Reference		Reference		Reference	
Yes	0.04 (–0.01, 0.08)	0.11	0.03 (0.01, 0.05)	0.02	0.03 (0.01, 0.06)	0.01
**§Prospective Memory**						
	**OR (95% CI)**	*p*	**OR (95% CI)**	*p*	**OR (95% CI)**	*p*
No	Reference		Reference		Reference	
Yes	0.75 (0.46, 1.22)	0.24	0.78 (0.60, 1.02)	0.07	0.84 (0.64, 1.10)	0.20

*Results from Model 3: adjusted for age, sex, smoking, Townsend deprivation index, education, income, employment status, home-ownership, self-reported health, alcohol intake, water intake, fish intake, red meat intake, fruit intake, vegetable intake, waist-to-hip ratio, physical activity, fasting time, coffee intake, and tea intake.

§Positive beta-coefficients for FI and OR > 1 for PM correspond to higher performance compared to non-consumers.

¶Negative beta-coefficients for Pairs and RT correspond to higher performance compared to non-consumers.

Table S14. Associations Between Recent Caffeine Drinking and Cognitive Function Tests Among White Participants Stratified by rs2472297 genotype ($p > 0.05$ for all interactions)*

	**CC**		**TC**		**TT**	
**Recent Caffeine**	**β (95% CI)**	***p***	**β (95% CI)**	***p***	**β (95% CI)**	***p***
**§Fluid Intelligence**						
No	Reference		Reference		Reference	
Yes	–0.06 (–0.24, 0.12)	0.51	–0.14 (–0.34, 0.06)	0.17	–0.23 (–0.73, 0.28)	0.38
**¶Reaction Time**						
No	Reference		Reference		Reference	
Yes	–5.14 (–8.71, –1.57)	0.005	–7.73 (–11.77, –3.68)	0.0002	–3.68 (–12.90, 5.55)	0.43
**¶Pairs Matching**						
No	Reference		Reference		Reference	
Yes	0.02 (–0.003, 0.04)	0.09	0.05 (0.02, 0.07)	0.0001	0.03 (–0.03, 0.08)	0.36
**§Prospective Memory**						
	**OR (95% CI)**	***p***	**OR (95% CI)**	***p***	**OR (95% CI)**	***p***
No	Reference		Reference		Reference	
Yes	0.86 (0.67, 1.10)	0.22	0.78 (0.60, 1.03)	0.08	0.62 (0.32, 1.19)	0.15

*Results from Model 3: adjusted for age, sex, smoking, Townsend deprivation index, education, income, employment status, home-ownership, self-reported health, alcohol intake, water intake, fish intake, red meat intake, fruit intake, vegetable intake, waist-to-hip ratio, physical activity, fasting time, coffee intake, and tea intake.

§Positive beta-coefficients for FI and OR > 1 for PM correspond to higher performance compared to non-consumers.

¶Negative beta-coefficients for Pairs and RT correspond to higher performance compared to non-consumers.

Table S15. Associations Between Recent Caffeine Drinking and Cognitive Function Tests Among White Participants Stratified by rs762551 (*p* > 0.05 for all interactions)*

	CC		AC		AA	
**Recent Caffeine**	**β (95% CI)**	***p***	**β (95% CI)**	***p***	**β (95% CI)**	***p***
**§Fluid Intelligence**						
No	Reference		Reference		Reference	
Yes	0.07 (–0.43, 0.56)	0.80	–0.17 (–0.38, 0.04)	0.12	–0.09 (–0.27, 0.08)	0.31
**¶Reaction Time**						
No	Reference		Reference		Reference	
Yes	–8.33 (–18.13, 1.46)	0.10	–4.84 (–8.94, –0.73)	0.02	–6.71 (–10.22, –3.21)	0.0002
**¶Pairs Matching**						
No	Reference		Reference		Reference	
Yes	0.005 (–0.06, 0.07)	0.88	0.04 (0.01, 0.06)	0.01	0.03 (0.01, 0.05)	0.003
**§Prospective Memory**						
	**OR (95% CI)**	***p***	**OR (95% CI)**	***p***	**OR (95% CI)**	***p***
No	Reference		Reference		Reference	
Yes	0.78 (0.41, 1.47)	0.45	0.93 (0.69, 1.27)	0.61	0.72 (0.57, 0.91)	0.01

*Results from Model 3: adjusted for age, sex, smoking, Townsend deprivation index, education, income, employment status, home-ownership, self-reported health, alcohol intake, water intake, fish intake, red meat intake, fruit intake, vegetable intake, waist-to-hip ratio, physical activity, fasting time, coffee intake, and tea intake.

§Positive beta-coefficients for FI and OR > 1 for PM correspond to higher performance compared to non-consumers.

¶Negative beta-coefficients for Pairs and RT correspond to higher performance compared to non-consumers.
